# *Drosophila* PRL-1 Is a Growth Inhibitor That Counteracts the Function of the Src Oncogene

**DOI:** 10.1371/journal.pone.0061084

**Published:** 2013-04-08

**Authors:** Krystle T. Pagarigan, Bryce W. Bunn, Jake Goodchild, Travis K. Rahe, Julie F. Weis, Leslie J. Saucedo

**Affiliations:** 1 Department of Biology, University of Puget Sound, Tacoma, Washington, United States of America; 2 Division of Basic Sciences, Fred Hutchinson Cancer Research Center, Seattle, Washington, United States of America; University of Dayton, United States of America

## Abstract

Phosphatase of Regenerating Liver (PRL) family members have emerged as molecular markers that significantly correlate to the ability of many cancers to metastasize. However, contradictory cellular responses to PRL expression have been reported, including the inhibition of cell cycle progression. An obvious culprit for the discrepancy is the use of dozens of different cell lines, including many isolated from tumors or cultured cells selected for immortalization which may have missing or mutated modulators of PRL function. We created transgenic *Drosophila* to study the effects of PRL overexpression in a genetically controlled, organismal model. Our data support the paradigm that the normal cellular response to high levels of PRL is growth suppression and furthermore, that PRL can counter oncogenic activity of Src. The ability of PRL to inhibit growth under normal conditions is dependent on a CAAX motif that is required to localize PRL to the apical edge of the lateral membrane. However, PRL lacking the CAAX motif can still associate indiscriminately with the plasma membrane and retains its ability to inhibit Src function. We propose that PRL binds to other membrane-localized proteins that are effectors of Src or to Src itself. This first examination of PRL in a model organism demonstrates that PRL performs as a tumor suppressor and underscores the necessity of identifying the conditions that enable it to transform into an oncogene in cancer.

## Introduction

In the past decade, Phosphatase of Regenerating Liver (PRL) family members have been touted as molecular markers that significantly correlate to the ability of cancers to metastasize [1],[2],[3]. In addition, laboratory studies indicate that PRLs are promising therapeutic targets; interfering with PRL function using antibodies and RNA interference has shown dramatic reduction in tumor formation in mice [4],[5]. PRL-1 was first isolated as a novel tyrosine phosphatase that is immediately transcribed following a partial hepatectomy, continually expressed in a number of tumor cell lines and able to transform non-tumorigenic cells [6],[7]. Later, PRL-2 and PRL-3 were identified by sequence analysis [8]. Studies in cell culture indicate that exogenous expression of PRLs can induce cell proliferation [7],[9],[10],[11], migration [12],[13],[14], and invasiveness [12],[11],[14]. Most significantly, constitutive expression of PRL-1 and -3 enable cultured cells to form tumors when injected into mice [12],[15],[13]. The potential of increased levels of PRLs to actively contribute to oncogenesis complements dozens of studies correlating PRL expression to tumor aggressiveness. PRL-3 first gained notoriety as a marker for metastasis when the Vogelstein lab found PRL-3 levels highly elevated in 100% of colon cancer metastases as compared to nonmetastatic tumors and normal colon epithelial [16]. Subsequent studies have corroborated PRL-3's association with colon cancer metastases [17],[18],[19],[20],[21] and extended the correlation between PRL-3 expression and metastasis of several other cancers, including liver [22],[23], [24], gastric [25],[26],[27],[28], breast [29],[30],[31], ovarian [32],[33], cervix [34], rectal [35], nasopharyngeal [36], esophageal [37],[38] and oral squamous cell [39].

In contrast, a few studies failed to support a positive relationship between PRLs and cancer; one study found that PRL-3 levels did not affect outcomes of ovarian cancer [40] and another study demonstrated that a 10-fold reduction in levels of PRL-3 correlated to lung cancer metastasis [41]. Failure to demonstrate the ability of PRL-3 to serve as an independent prognostic factor led Hatate *et al.* [42] to speculate that PRL-3 expression may not represent a direct causative mechanism of liver metastasis. Surprisingly, PRL-3 was isolated as a p53 target that contributed to the cell cycle arrest of damaged cells [43]. Additional studies also demonstrated PRL-3 to halt cell cycle progression when exogenously introduced into non-damaged cells. However, the ability of PRL-3 to inhibit cell cycle progression was not universal, occurring in three of five cell lines tested [43]. The authors hypothesized that the discrete responses likely reflect existing mutations in the various tumor cell lines that alter downstream effectors of PRL-3. Because their initial findings were from primary mouse embryo fibroblasts, the ability for PRL-3 to suppress cell growth may be its “normal” function. *In vivo* expression surveys support the notion that PRLs can contribute to growth arrest. For example, PRL-1 is highly expressed in differentiated intestinal cells relative to undifferentiated counterparts [44]. In addition, Kong et al. [45] showed that PRL-1 expression correlates with terminal differentiation of other epithelial tissues, such as the kidney and lung. PRL-2 and -3 can also associate with differentiated tissues, with both preferentially expressed in muscle tissue [8].

All three PRL family members contain a consensus tyrosine phosphatase domain and a C-terminal prenylation, CAAX motif [7],[8]. Only two proteins have been shown to be directly dephosphorylated by PRL: Ezrin [46] and a poorly characterized basic leucine zipper (bZIP) protein called ATF-7 [47]. However, in all cases examined, a catalytically active phosphatase domain was required for phenotypes resulting from PRL-3 overexpression, including increases in proliferation [9], migration [12],[13],[48] and metastases formation in animal models [15]. Another important regulator of PRL function is farnesylation of the CAAX motif. Either mutating the motif or adding a farnesyltransferase inhibitor leads to subcellular redistribution of PRLs, from membrane to nucleus [49],[50]. This relocalization results in a block to cellular responses to ectopic PRL expression, such as enhanced proliferation [50], migration [48],[50], and metastasis [51]. However, another group determined that cytoplasmic localization is positively related to metastasis of cervical cancer [34], confounding a direct relationship between PRL subcellular localization and cellular outcome.

Two signal transduction pathways that have been implicated as oncogenic effectors of PRLs are Src and PI3K signaling. PRL-3 activates Src signaling [11], [52],[53] by reducing the synthesis of protein, Csk, an inhibitor of the pathway [11],[54] and upregulation of PRL1 activates the Src kinase through increased Tyr416 phosphorylation and cell migration [53]. Similar to its effect on Src signaling, PRL-3 promotes PI3K signaling by reducing levels of a protein that normally antagonizes the pathway, in this case, PTEN [55]. This results in activation of Akt [55], which is well established as protecting cells against apoptosis and also promoting cell migration [56],[57]. Interestingly, inhibition of Akt has also been shown to be a key player for PRL-3 to arrest cells [43]. Experimenting with levels of PRL-3 overexpression appears to reconcile the opposing effects of PRL-3 on Akt; Basak *et al.*, (2008) could detect activation of Akt in response to PRL-3, but only transiently, until level of PRL-3 became highly elevated.

Although there is a rapidly growing amount of literature on the mammalian family of PRL phosphatases, several studies have conflicting results. These studies each examine PRL in a different genetic environment, which may mean modulators and effectors of PRL localization or function are missing or mutated. Our study using *Drosophila* is the first to examine overexpressed PRL in genetically controlled animal model. This system confirms that PRL can function as a growth inhibitor under normal and oncogenic conditions that can be dependent on submembrane distribution.

## Results

### Drosophila PRL-1 inhibits growth

**Figure 1:**
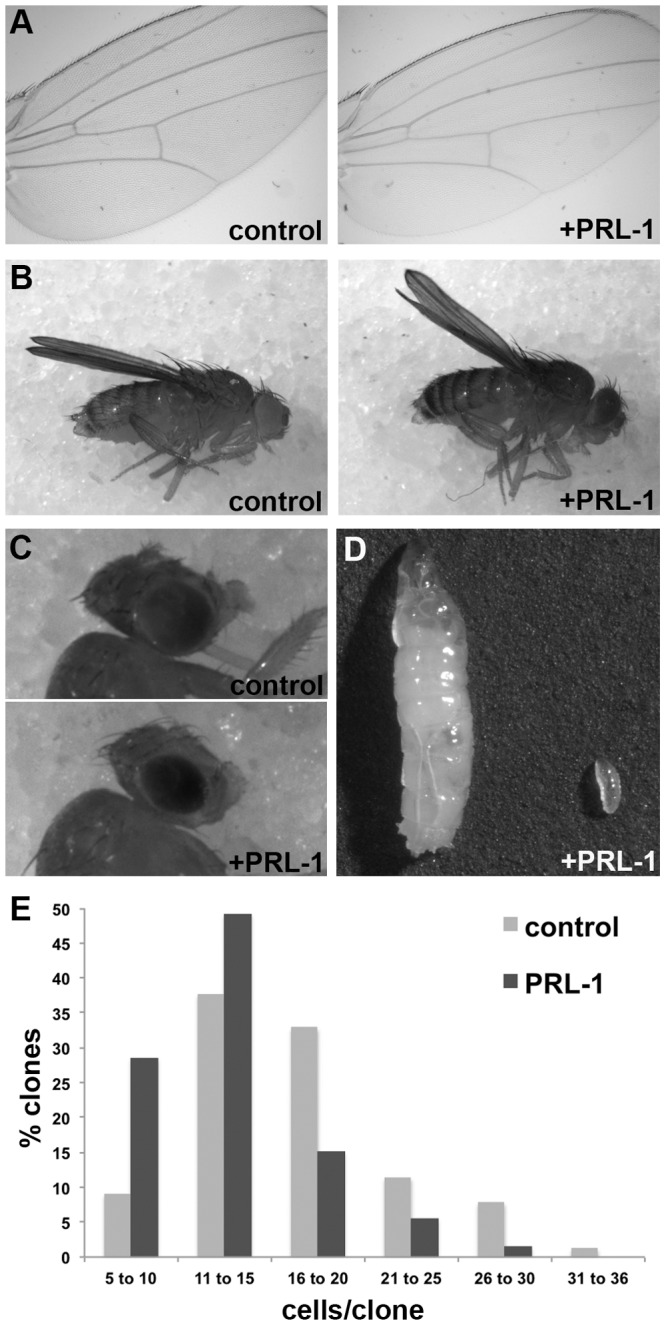
Overexpression of dPRL-1 inhibits growth. Expression of dPRL-1 in the posterior compartment of the wing (*w; en-Gal4/UAS-dPRL-1; +*) reduces surface area by 20% (A) whereas expression in the dorsal compartment of the wing (*w; ap-Gal4, UAS-dPRL-1; +*) leads to an upward curvature (B). Expression in developing eyes (*ey-flp; UAS-dPRL-1; act>CD2>Gal4*) reduces the size of the adult head (C) while constitutive expression (*w; UAS-dPRL-1; act-Gal4*) prevents larvae from gaining mass (D). Lastly, expression of dPRL-1 in clones of cells in the developing wing disc (*hs-flp; UAS-dPRL-1; act>CD2>Gal4*) reduced proliferation (D).

The *Drosophila* genome encodes a single PRL protein (dPRL-1), which is highly similar (74–76%) to all three human PRLs and contains the three domains shown to be required for PRL function in mammals: a Dual Specific Phosphatase (DSP) active site (HCxxGxxR) [9],[12],[13],[15],[48], an aspartate (Asp77) that has been demonstrated to facilitate phosphate transfer [58], and a C-terminal, membrane-targeting CAAX motif adjacent to a polybasic region [48]–[51]. Transgenic animals containing full-length dPRL-1 under the control of Upstream Activating Sequences (UAS) were crossed to numerous lines of animals that expressed the transcriptional activator GAL4 in a tissue-specific manner. Overexpression of dPRL-1 broadly resulted in inhibition of growth that in some instances resulted in lethality. For example, expression in the developing larval wing decreased tissue size in the adult; expression in the posterior compartment of the wing using *engrailed-Gal4* (*en-Gal4*) reduced the surface area by 20% (p = 0.003, Figures 1A, S1) while expression in the dorsal compartment using *apterous-Gal4* (*ap-Gal4*) lead to an upward curvature, also indicative of a decrease in surface area (Figure 1B). Similarly, expression of dPRL-1 in the developing eye using the *eyeless* flip-out system (*ey-flp; +; act>CD2>Gal4*) led to a smaller eye and head capsule (Figure 1C). Finally, ubiquitous expression of dPRL-1 using *actin-Gal4* (*act-Gal4*) prevented larva growth; although the larvae consumed food, most stalled in the first instar (L1) of development (Figure 1D) for 2–6 days before dying. Dissection of the animals did not reveal any obvious morphological defects. Generating random clones in developing wings [58],[59] enabled us to determine that overexpression of dPRL-1 reduced the average clone size from 17.9+/−0.58 cells to 13.4+/−0.39 cells (p<0.0001, n = 120 each genotype, Figure 1E). Because co-expression of apoptosis inhibitors (p35 and DIAP) and caspase staining indicated that the reduction in tissue growth was not due to apoptosis (data not shown), we conclude that the reduced size of clones overexpressing dPRL-1 was due to an 11% increase in cell doubling time (CDT [59]).

### dPRL-1 is ubiquitously expressed and localizes to both the cytoplasm and plasma membrane

**Figure 2:**
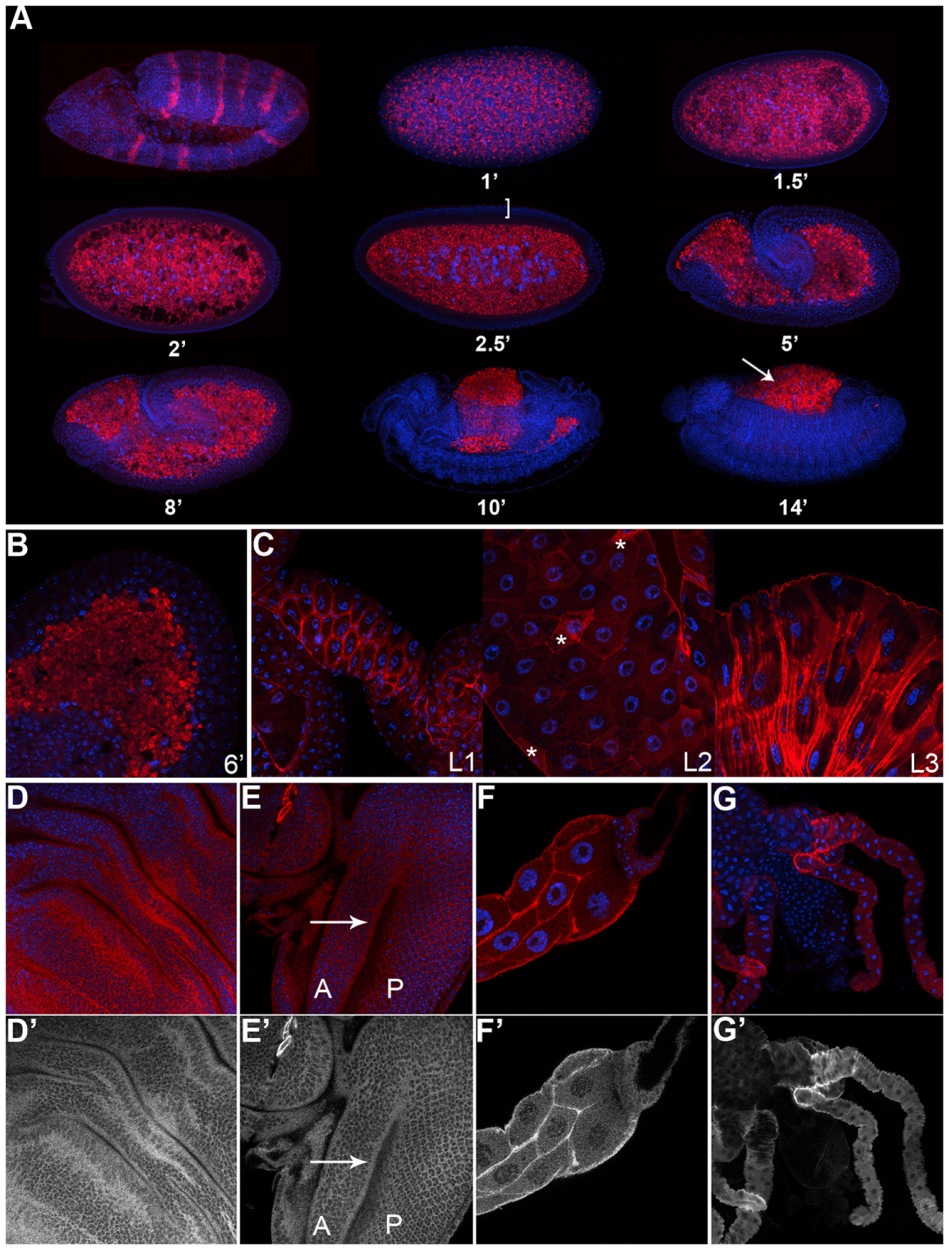
Endogenous dPRL-1 levels and localization throughout *Drosophila* development. (A) Immunodetection of dPRL-1 (red) during embryo development. Directing dPRL-1 under the control of engrailed (*w; en-Gal4/UAS-dPRL-1*) demonstrates specificity of antibody (top, left corner) while subsequent images demonstrate that endogenous dPRL-1 is located in cytoplasmic compartments from 1 to 14 hours after egg laying. The bracket marks the location of first cellularization of the blastoderm, where individual cells first form and the arrow highlights the amnioserosa. (B) Higher magnification (600×) of dPRL-1 cytoplasmic localization during nascent cell formation. (C–G) dPRL-1 expression is cytoplasmic and membranous in various third instar larval tissues. (C) dPRL-1 in the midintestine throughout larval development (L1→L3) show the most variation in cytoplasmic staining (those with higher levels are indicated by asterisk). A selection of additional larval tissues— the wing disc (D–D'), eye/antenna disc (E–E', with morphogenetic furrow indicated by arrow), salivary gland (F–F') and proventriculus/gastric caecum (G–G') all shown. Blue staining marks nuclei and gray staining (D–G) is dPRL-1.

To examine when and where dPRL function may function *in vivo*, we monitored dPRL-1 subcellular localization throughout *Drosophila* embryogenesis and larval development. By expressing dPRL-1 under the control of an engrailed promoter, we verified that our dPRL-1 antibody was functional by observing high levels of dPRL-1 protein in the posterior compartments of the embryo epidermis (Figure 2A). Prior to cellularization, dPRL-1 is evenly expressed throughout the syncytium (Figure 2A). Following cellularization, dPRL-1 levels are relatively low in the newly formed blastoderm, but can be seen in the cytoplasm (Figure 2A,B). As embryogenesis proceeds, dPRL-1 remains ubiquitously and cytoplasmically expressed, though most abundant in the amnioserosa in later stages of embryogenesis (Figure 2A). Analysis of the first through third larval instar tissues showed that dPRL-1 becomes localized to and more abundant at the plasma membrane though cytoplasmic staining is still detected (Figure 2C–G). The larval midgut demonstrated the most dynamic expression, with some cells showing predominant dPRL-1 staining at plasma membrane and others showing very high levels of dPRL-1 in the cytoplasm (Figure 2C). dPRL-1 appears to be ubiquitously expressed throughout larval development although with variable levels; the gastric caecum consistently demonstrated very strong staining for dPRL-1 (Figure 2G), while the larval brain was consistently among the lowest (data not shown). In the developing eye and wing discs (the tissues used for adult analysis of dPRL-1 function) dPRL-1 is most abundant at the plasma membrane (Figure 2D–E). Staining in the developing eye (Figure 2E) demonstrates that dPRL-1 levels and localization are similar in both actively dividing cells (anterior to the morphogenetic furrow) and differentiated cells (posterior to the morphogenetic furrow). Thus, in concordance with mammalian studies [44],[45], dPRL-1 expression alone does not serve as an indicator of cell proliferation.

### The CAAX domain is needed for growth inhibition and submembrane localization of dPRL-1

**Figure 3:**
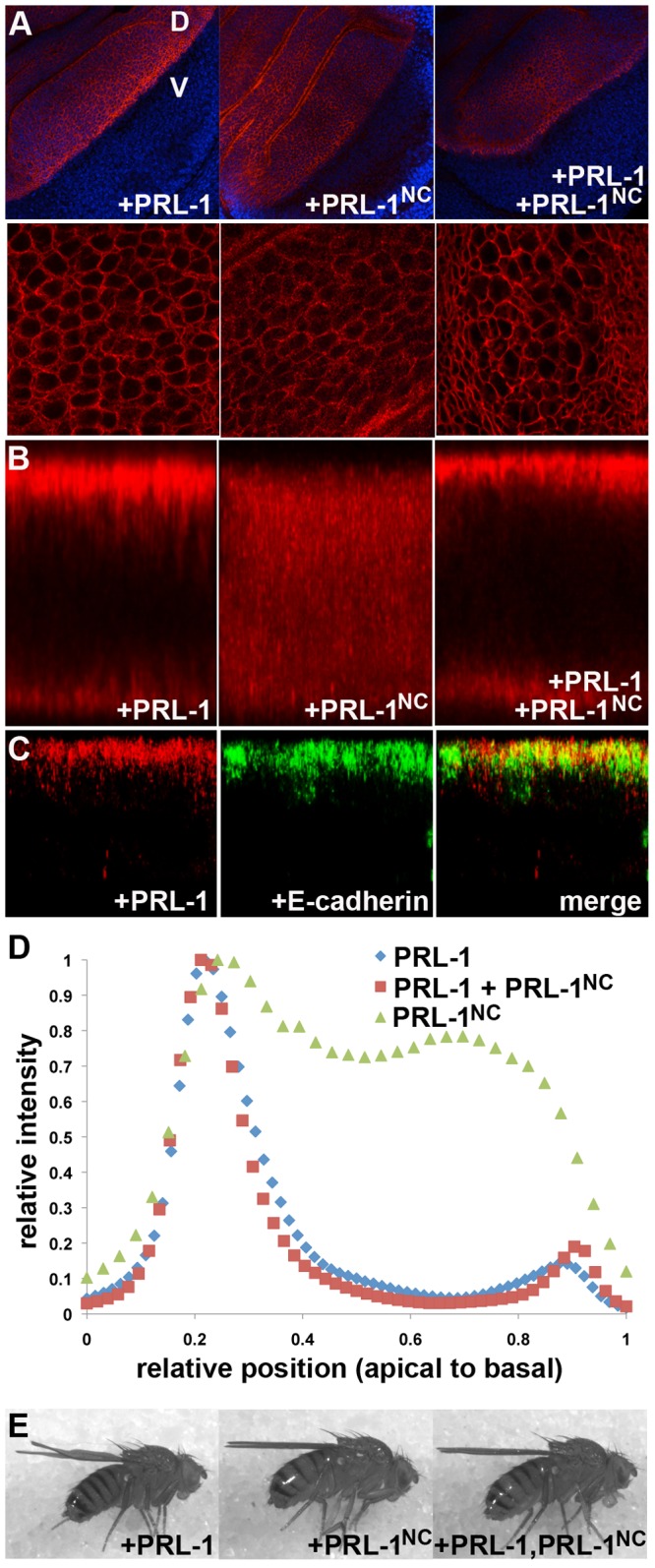
CAAX motif required for PRL-1 localization and function. Immunodetection of dPRL-1 (red) expressed in the dorsal compartment of the wing (*w; ap-GAL4, UAS-dPRL-1; +*) indicates that removal of the CAAX motif allows dPRL-1 to remain associated with the membrane (A, middle panels) but that it is no longer concentrated at the apical edge of epithelia (B, middle panel). (C) Co-staining with E-cadherin (green) indicates some overlap in dPRL-1 and E-cadherin localization. (D) Quantification of relative levels and position of dPRL-1 shows that co-expression of dPRL-1 and dPRL-1^NC^ resumes a restricted distribution although the ability of dPRL-1 to suppress growth is compromised (E).

Endogenous dPRL-1 is primarily localized to the plasma membrane in epithelial cells of developing larva, and this subcellular localization held true under conditions of overexpression that led to growth inhibition (Figure 3A). Past reports have indicated that the C-terminal CAAX motif is a requirement for the addition of a farnesyl “tail” to anchor mammalian PRLs to the membrane [48]–[51]. In order to determine the role of the CAAX motif in both localization and function of dPRL-1, we created transgenic animals lacking the four, terminal amino acids. Surprisingly, the modified dPRL-1^NC^ still localized to the plasma membrane, although qualitatively, it appeared less tightly associated (Figure 3A). Because developing wing epithelia are pseudostratified, we used Z-section analysis to more closely examine dPRL-1's subcellular distribution. This analysis indicated that wild-type dPRL-1 was found on the lateral side of epithelial cells, but was primarily restricted (>80% of total signal) towards the apical ends (Figure 3B,D). Co-staining with overexpressed E-cadherin partially overlap, indicating that dPRL-1 may interact with components of adherens junctions (Figure 3C). In contrast, dPRL-1^NC^ showed relatively uniform distribution on the lateral sides with only a slight peak in apical intensity overlapping with dPRL-1 (Figure 3B,D). This disruption in how dPRL-1 associates with the plasma membrane had functional consequences; dPRL-1^NC^ failed to inhibit growth (Figure 3E.) Interestingly, when both transgenes were expressed, the organismal phenotype of dPRL-1^NC^ dominated; growth inhibition by wild-type dPRL-1 was suppressed (Figure 3E), even though the majority of dPRL-1 was properly localized (Figure 3A,B,D). This data suggests that that dPRL-1 forms homo-quaternary structures, a model that is supported by *in vitro* studies using mammalian PRL-1 [60],[50]. Interactions between dPRL-1 and dPRL-1^NC^ could enable a complex to localize properly via the intact CAAX motif of dPRL-1 but disrupt function if the dPRL-1^NC^ incorporated into the complex without a farnesyl group to orient it accurately.

### dPRL-1 counters Src oncogene phenotypes

**Figure 4:**
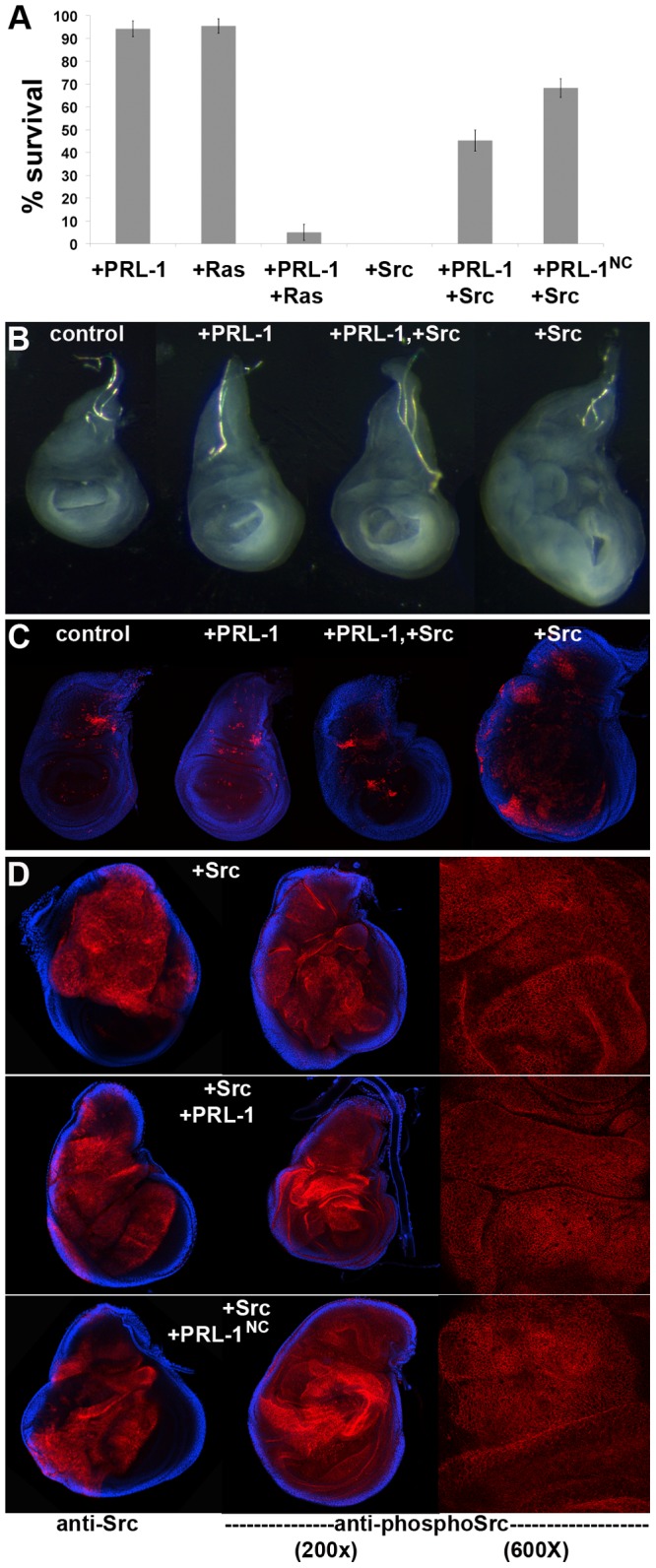
dPRL-1 counters Src-induced lethality and overgrowth. (A) Overexpression of Src in the dorsal compartment(s) of developing larva (*w; ap-Gal4; UAS-Src*) results in adult lethality, which is strongly rescued by co-expression of dPRL-1 or dPRL-1^NC^. In contrast, overexpression of Ras in the dorsal compartment (*ap-GAL4, UAS-Ras*) resulted in lethality during pupal stages, which was accelerated by co-expression of dPRL-1. Data is presented as average +/− standard error of viable adults for animals overexpressing Src or viable prepupae for animals overexpressing Ras. Larval wings overexpressing Src are grossly overgrown (B) and have elevated levels of apoptosis (C); both phenotypes are suppressed by coexpression of dPRL-1. (D) Total Src protein (anti-Src) or activated Src (anti-phosphoSrc) are both elevated in animals overexpressing Src (top panels). Co-expression of dPRL-1 or dPRL-1^NC^ do not affect the levels, activation or localization of Src (middle, bottom panels).

We used the curved wing phenotype resulting from expression of dPRL-1 in the dorsal compartment using *ap-Gal4* of the wing to identify genetic interactions with known oncogenes. Surprisingly, we found that overexpression of Src or Ras resulted in lethality; both oncogenes preventing pupae from eclosing. dPRL-1 co-overexpressing significantly suppressed Src-induced lethality, enabling 45% of expected adults to eclose. In contrast, dPRL-1 co-overexpression accelerated lethality resulting from overexpression of Ras; preventing animals from pupariation (Figure 4A). Investigation of the developing wings of these animals showed that overexpression of Src led to massive overgrowth and developmental disorganization (Figure 4B), which was suppressed by co-overexpression of dPRL-1 (Figure 4B). Although wings from animals overexpressing Ras and dPRL-1 also appeared smaller than those overexpressing Ras alone, this finding was confounded by the larvae also being smaller (data not shown). Larvae expressing Ras and dPRL-1 also seemed lethargic, indicating the lethal phenotype likely results from expression in a tissue besides the wing. Therefore, we focused our attention on Src. To investigate whether this suppression in Src-induced tissue growth was due to growth inhibition by dPRL-1 or via an induction of apoptosis, developing wings were stained for cleaved, caspase 3 (Fig. 4C). Wings overexpressing only Src demonstrated the highest levels of apoptosis, even beyond the dorsal compartment, perhaps as an organismal response to massive overgrowth. Wings overexpressing dPRL-1 in conjunction with Src had levels of activated caspase 3 similar to controls, thus supporting the model that PRL-1 counters Src-induced overgrowth by slowing cell division rather than by increasing apoptosis. However, the CAAX motif of dPRL-1 was not required to overcome Src-induced lethality (Figure 4A). This finding suggests that the mechanism to counter Src is separate from dPRL-1's ability to inhibit growth under normal conditions, which is dependent on the CAAX motif. Similar increases of total and phospho-activated Src in the membranes of developing wing epithelia with or without co-expression of dPRL-1/dPRL-1^NC^ indicated that dPRL-1/dPRL-1^NC^ do not directly affect Src activity (Fig. 4D).

## Discussion

dPRL-1 is a ubiquitously expressed protein found in both proliferating and differentiated tissues of *Drosophila* (Figure 2) that can function as a growth inhibitor at elevated levels (Figure 1). Our work supports the model that other cellular alterations are required for elevated levels of PRL to promote cancer [43]. For example, because the CAAX motif is required for dPRL-1 to suppress growth (Figure 3E), cellular modifications that interfere with the motif could be one means towards enabling PRLs to act as oncogenes instead. Indeed, our analysis of endogenous dPRL-1 expression during embryogenesis demonstrated that dPRL-1 levels can be high in the cytoplasm (Figure 2A,C) in spite of an intact CAAX motif, suggesting other proteins can override CAAX-driven membrane localization. While others' work has highlighted the need of the CAAX motif for PRLs function [48]–[51], we are the first to see that at least one member of the PRL family can still associate with the plasma membrane without CAAX (Figure 3A). This association may occur through the polybasic region adjacent to CAAX, which has been shown to be required for membrane association in addition to CAAX [50]. Our work is also the first to report the accumulation of a PRL family member to apico-lateral locations in epithelial cells; suggesting that dPRL-1 is forming stable interactions with other membrane-bound proteins.

Intriguingly, we found that elevated levels of dPRL-1 can have opposing outcomes in genetic backgrounds expressing known oncogenes; resulting in synergistic lethality with Ras but rescuing Src-induced lethality (Figure 4A). Src overexpression likely results in lethality because the massively overgrown wing disc (which contributes to both the adult wing and thorax) becomes developmentally disorganized. While dPRL-1 effectively inhibits Src-induced overgrowth (Figure 4B,C), another mechanism to counter Src function must exist because dPRL-1^NC^, which does not inhibit growth under normal levels of Src (Figure 3E), retains the ability to counter Src-induced lethality (Figure 4A). One possibility was that dPRL-1/dPRL-1^NC^ could increase apoptosis, thus eliminating excess tissue. Furthermore, this phenotype could be accomplished by dPRL-1 leading to an increase in Src activity as has been seen in mammalian studies [11],[52],[53]. Previous studies in *Drosophila* have shown a dose response with lower levels of Src leading to proliferation but higher levels resulting in apoptosis [61]. However, we did not detect elevated levels of apoptosis in animals overexpressing both dPRL-1 and Src (Figure 4C). The more straightforward model of dPRL-1 simply countering activation of Src was also not supported by our studies (Figure 4D). Because dPRL-1/dPRL-1^NC^ and Src are both membrane localized (Figure 4D), we suspect dPRL-1/dPRL-1^NC^ may physically interfere with either Src or an effector of Src function.

While dPRL-1s ability to inhibit growth is in concordance with one report from the mammalian literature [43], there are certainly differences to highlight between *Drosophila* and mammalian studies. Sequence analysis shows that the aspartate, that serves as a proton donor is present in *Drosophila* but not in the context of the WPD loop, as seen in mammalian PRL family members [62]. While this aspartate is also not found in WPD loop in other PTPs like VHR, cdc14, and PTEN [62], it may point to different substrates between mammals and flies. In addition, catalytic activity of mammalian PRL1 is regulated by the redox environment [63],[64],[65], and thought to exist in an inactive conformation under normal cellular conditions [65]. Possibly, differences in redox regulation between *Drosophila* and cultured mammalian cells could account for differing outcomes in response to PRL-1 overexpression. For example, altered redox environments in transformed cells could switch PRLs to an abnormal, catalytically active state. Another important difference between *Drosophila* and mammals may be the p53 network. While supporting the model that PRL-3 is a transcriptional target of p53, Min *et al.* [66],[67] report that PRL-3 then functions in a negative, autoregulatory loop by decreasing levels of p53, which would help transform cells. They identify MDM2 and PIRH2 as the important players in this pathway; but since neither protein is found in *Drosophila*, this oncogenic path is not conserved. In spite of the differences between mammals and *Drosophila*, flies have successfully informed numerous mechanisms that contribute to human cancer biology [68]. We have established a new system that has revealed novel characteristics of the PRL family and will help decipher the role(s) PRLs play in cancers.

## Materials and Methods

### *Drosophila* lines

Full-length cDNA of *dPRL-1* (LD12894, BDGP) was cloned into pUAST via KpnI and XbaI restriction sites and sequenced, confirming a wild type sequence that encoded the expected protein of 176 amino acids (Accession #NP_609780). Transgenic animals were produced by BestGene (Chino Hills, CA). In order to construct a modified dPRL-1 lacking its CAAX motif, PCR primers were developed that amplified a truncated version of *dPRL-1* (LD12894), while introducing restriction sites (EagI and KpnI) for subsequent cloning into pUAST. The primers sequences were forward primer: 5′-ATCGGCCGATGAGCATCACCATGCGTC-3′ reverse primer 5′- TAGGTACCCTATGAATTCTTATGACCATT-3′ (both primers from Invitrogen- Carlsbad, CA). Following sequence confirmation that the CAAX motif had been deleted, transgenic animals were produced by GenetiVision (Houston, TX). Other fly stocks were: w^118^; +; +, yw; enGal4; + (Bloomington stock #6356), w; apGal4/CyOGFP (Bloomington stock #3041), eyFLP (Bloomington stock #8205), w;+;act>cd2>Gal4, UASGFP_NLS_ [59], MAESrc (Bloomington stock #7342), UAS-DECad [69].

### Immunohistochemistry

Polyclonal antibodies to dPRL-1 were generated in rabbits using a peptide consisting of amino acids 158–176 of dPRL-1 (Open Biosystems, Huntsville, AL). Other primary antibodies used were: anti-cleaved Caspase (Cell Signaling), anti-DE-Cadherin (DHSB), anti-Src64CT and anti-pY343Src [70]. Embryos and larval tissues were fixed in 4% formaldehyde (EM Biosiences) in PBS prior to staining. Primary antibodies were diluted in PBS, 0.1% Triton X-100, 1% BSA at various dilutions: 1∶50 (DE-Cadherin), 1∶100 (PRL-1, pY343Src), 1∶400 (cleaved Caspase) and 1∶5,000 (Src64CT). Cy3- (Invitrogen) and CF488-conjugated (sigma) secondary antibodies were diluted 1∶2000 in PBS, 0.3% Triton X-100, 0.1% BSA, 0.1% Na-N_3_. Tissues were mounted in a 1∶1 solution of PBS and Vectashield mounting medium with DAPI (Vector Labs). Microscopy was conducted using an Olympus BX40 Laboratory Microscope connected to a Lumen Dynamics X-Cite© Series 120Q Fluorescence Microscopy Illumination System and a ProgRes© C3 Jenoptik Digital Microscope Camera. Images were captured using the ProGres© Mac Capture Pro program. Confocal microscopy was conducted using a Nikon A1 Confocal Laser Microscope system. Images were captured and signal intensity quantified using the EZ-C1 and NIS-Elements Basic Research 3.10 software. Z-stacks were taken with step size 0.35 µm and pixel dwell 1.68 µs. Signal intensities were collected in flat portions of the dorsal (experimental) and ventral (control) compartments of wing discs. The average pixel intensity of two matched optical sections were examined per sample. High laser power coupled with minimal gain settings were used to achieve the strongest signal to noise ratios (SNR) in the absence of photo-bleaching.

Images were prepared using Adobe Photoshop CS5 Extended.

### Tissue and cell growth analyses Generation of clones/CDT

To compare tissue sizes in the posterior compartment, the Lasso and Histogram function (total pixels) of Adobe Photoshop were used to quantify the surface area of tissue between the L5 vein and wing margin. As an internal control, anterior surface area was quantified between the L1 and L2 veins. Clones overexpressing PRL-1 were created by the Flp-out technique [58],[59] by applying heat shock for 6″ at 37°C 48 hours prior to wandering.

### Viability assay

Crosses were set in embryo collection chambers, and adults were left to lay on grape plates for 2 hours. Using GFP as a marker for control (GFP+) and experimental (GFP−), L1 larvae were placed into vials and genotypes of pupae (for Ras assays) and adults (for Src assays) were tallied.

## Supporting Information

Figure S1 **Quantification of growth inhibition following dPRL-1 expression in the wing.** Comparison of surface area in the posterior (P) and anterior (A) compartments of adult wings of animals expressing dPRL-1 (*w; enGal4, UAS-dPRL-1*) compared to control (*w; enGal4*; +). dPRL-1 reduces the area of the posterior compartment by 20% (p = 003). The small reduction in the anterior compartment was not statistically significant (p = 0.24). Data is presented as average +/− standard error.
(TIF)

## References

[1] Stephens BJ, Han H, Gokhale V, Von Hoff DD (2005) PRL phosphatases as potential molecular targets in cancer. Mol Cancer Ther 4: 1653–1661 doi:10.1158/1535-7163.MCT-05-0248 16275986 10.1158/1535-7163.MCT-05-0248

[2] Bessette DC, Qiu D, Pallen CJ (2008) PRL PTPs: mediators and markers of cancer progression. Cancer Metastasis Rev 27: 231–252 doi:10.1007/s10555-008-9121-3 18224294 10.1007/s10555-008-9121-3

[3] Al-Aidaroos AQO, Zeng Q (2010) PRL-3 phosphatase and cancer metastasis. J Cell Biochem 111: 1087–1098 doi:10.1002/jcb.22913 21053359 10.1002/jcb.22913

[4] Li Z, Zhan W, Wang Z, Zhu B, He Y, et al (2006) Inhibition of PRL-3 gene expression in gastric cancer cell line SGC7901 via microRNA suppressed reduces peritoneal metastasis. Biochem Biophys Res Commun 348: 229–237 doi:10.1016/j.bbrc.2006.07.043 16875667 10.1016/j.bbrc.2006.07.043

[5] Guo K, Tang JP, Tan CPB, Wang H, Zeng Q (2008) Monoclonal antibodies target intracellular PRL phosphatases to inhibit cancer metastases in mice. Cancer Biol Ther 7: 750–757. 18364570 10.4161/cbt.7.5.5764

[6] Mohn KL, Melby AE, Tewari DS, Laz TM, Taub R (1991) The gene encoding rat insulinlike growth factor-binding protein 1 is rapidly and highly induced in regenerating liver. Mol Cell Biol 11: 1393–1401. 1705004 10.1128/mcb.11.3.1393-1401.1991 PMC369411

[7] Diamond RH, Cressman DE, Laz TM, Abrams CS, Taub R (1994) PRL-1, a unique nuclear protein tyrosine phosphatase, affects cell growth. Mol Cell Biol 14: 3752–3762. 8196618 10.1128/mcb.14.6.3752-3762.1994 PMC358742

[8] Zeng Q, Hong W, Tan YH (1998) Mouse PRL-2 and PRL-3, two potentially prenylated protein tyrosine phosphatases homologous to PRL-1. Biochem Biophys Res Commun 244: 421–427 doi:10.1006/bbrc.1998.8291 9514946 10.1006/bbrc.1998.8291

[9] Matter WF, Estridge T, Zhang C, Belagaje R, Stancato L, et al (2001) Role of PRL-3, a human muscle-specific tyrosine phosphatase, in angiotensin-II signaling. Biochem Biophys Res Commun 283: 1061–1068 doi:10.1006/bbrc.2001.4881 11355880 10.1006/bbrc.2001.4881

[10] Werner SR, Lee PA, DeCamp MW, Crowell DN, Randall SK, et al (2003) Enhanced cell cycle progression and down regulation of p21(Cip1/Waf1) by PRL tyrosine phosphatases. Cancer Lett 202: 201–211. 14643450 10.1016/s0304-3835(03)00517-2

[11] Liang F, Liang J, Wang W-Q, Sun J-P, Udho E, et al (2007) PRL3 promotes cell invasion and proliferation by down-regulation of Csk leading to Src activation. J Biol Chem 282: 5413–5419 doi:10.1074/jbc.M608940200 17192274 10.1074/jbc.M608940200

[12] Zeng Q, Dong J-M, Guo K, Li J, Tan H-X, et al (2003) PRL-3 and PRL-1 promote cell migration, invasion, and metastasis. Cancer Res 63: 2716–2722. 12782572

[13] Wu X, Zeng H, Zhang X, Zhao Y, Sha H, et al (2004) Phosphatase of regenerating liver-3 promotes motility and metastasis of mouse melanoma cells. Am J Pathol 164: 2039–2054 doi:10.1016/S0002-9440(10)63763-7 15161639 10.1016/S0002-9440(10)63763-7 PMC1615773

[14] Wang Y, Lazo JS (2012) Metastasis-associated phosphatase PRL-2 regulates tumor cell migration and invasion. Oncogene 31: 818–827 doi:10.1038/onc.2011.281 21765462 10.1038/onc.2011.281 PMC4553949

[15] Guo K, Li J, Tang JP, Koh V, Gan BQ, et al (2004) Catalytic domain of PRL-3 plays an essential role in tumor metastasis: formation of PRL-3 tumors inside the blood vessels. Cancer Biol Ther 3: 945–951. 15326366 10.4161/cbt.3.10.1111

[16] Saha S, Bardelli A, Buckhaults P, Velculescu VE, Rago C, et al (2001) A phosphatase associated with metastasis of colorectal cancer. Science 294: 1343–1346 doi:10.1126/science.1065817 11598267 10.1126/science.1065817

[17] Bardelli A, Saha S, Sager JA, Romans KE, Xin B, et al (2003) PRL-3 expression in metastatic cancers. Clin Cancer Res 9: 5607–5615. 14654542

[18] Kato H, Semba S, Miskad UA, Seo Y, Kasuga M, et al (2004) High expression of PRL-3 promotes cancer cell motility and liver metastasis in human colorectal cancer: a predictive molecular marker of metachronous liver and lung metastases. Clin Cancer Res 10: 7318–7328 doi:10.1158/1078-0432.CCR-04-0485 15534108 10.1158/1078-0432.CCR-04-0485

[19] Peng L, Ning J, Meng L, Shou C (2004) The association of the expression level of protein tyrosine phosphatase PRL-3 protein with liver metastasis and prognosis of patients with colorectal cancer. J Cancer Res Clin Oncol 130: 521–526 doi:10.1007/s00432-004-0563-x 15133662 10.1007/s00432-004-0563-x PMC12161869

[20] Wang Y, Li Z-F, He J, Li Y-L, Zhu G-B, et al (2007) Expression of the human phosphatases of regenerating liver (PRLs) in colonic adenocarcinoma and its correlation with lymph node metastasis. Int J Colorectal Dis 22: 1179–1184 doi:10.1007/s00384-007-0303-1 17440740 10.1007/s00384-007-0303-1

[21] Xing X, Peng L, Qu L, Ren T, Dong B, et al (2009) Prognostic value of PRL-3 overexpression in early stages of colonic cancer. Histopathology 54: 309–318 doi:10.1111/j.1365-2559.2009.03226.x 19236507 10.1111/j.1365-2559.2009.03226.x

[22] Zhao W-B, Li Y, Liu X, Zhang L-Y, Wang X (2008) Evaluation of PRL-3 expression, and its correlation with angiogenesis and invasion in hepatocellular carcinoma. Int J Mol Med 22: 187–192. 18636172

[23] Xu Y, Zhu M, Zhang S, Liu H, Li T, et al (2010) Expression and prognostic value of PRL-3 in human intrahepatic cholangiocarcinoma. Pathol Oncol Res 16: 169–175 doi:10.1007/s12253-009-9200-y 19757198 10.1007/s12253-009-9200-y

[24] Kong L, Li Q, Wang L, Liu Z, Sun T (2007) The value and correlation between PRL-3 expression and matrix metalloproteinase activity and expression in human gliomas. Neuropathology 27: 516–521 doi:10.1111/j.1440-1789.2007.00818.x 18021371 10.1111/j.1440-1789.2007.00818.x

[25] Miskad UA, Semba S, Kato H, Yokozaki H (2004) Expression of PRL-3 phosphatase in human gastric carcinomas: close correlation with invasion and metastasis. Pathobiology 71: 176–184 doi:10.1159/000078671 15263806 10.1159/000078671

[26] Miskad UA, Semba S, Kato H, Matsukawa Y, Kodama Y, et al (2007) High PRL-3 expression in human gastric cancer is a marker of metastasis and grades of malignancies: an in situ hybridization study. Virchows Arch 450: 303–310 doi:10.1007/s00428-006-0361-8 17235563 10.1007/s00428-006-0361-8

[27] Ooki A, Yamashita K, Kikuchi S, Sakuramoto S, Katada N, et al (2009) Phosphatase of regenerating liver-3 as a prognostic biomarker in histologically node-negative gastric cancer. Oncol Rep 21: 1467–1475. 19424625 10.3892/or_00000376

[28] Dai N, Lu A-P, Shou C-C, Li J-Y (2009) Expression of phosphatase regenerating liver 3 is an independent prognostic indicator for gastric cancer. World J Gastroenterol 15: 1499–1505. 19322925 10.3748/wjg.15.1499 PMC2665146

[29] Radke I, Götte M, Kersting C, Mattsson B, Kiesel L, et al (2006) Expression and prognostic impact of the protein tyrosine phosphatases PRL-1, PRL-2, and PRL-3 in breast cancer. Br J Cancer 95: 347–354 doi:10.1038/sj.bjc.6603261 16832410 10.1038/sj.bjc.6603261 PMC2360632

[30] Wang L, Peng L, Dong B, Kong L, Meng L, et al (2006) Overexpression of phosphatase of regenerating liver-3 in breast cancer: association with a poor clinical outcome. Ann Oncol 17: 1517–1522 doi:10.1093/annonc/mdl159 16873432 10.1093/annonc/mdl159

[31] Hao R-T, Zhang X-H, Pan Y-F, Liu H-G, Xiang Y-Q, et al (2010) Prognostic and metastatic value of phosphatase of regenerating liver-3 in invasive breast cancer. J Cancer Res Clin Oncol 136: 1349–1357 doi:10.1007/s00432-010-0786-y 20140626 10.1007/s00432-010-0786-y PMC11827964

[32] Polato F, Codegoni A, Fruscio R, Perego P, Mangioni C, et al (2005) PRL-3 phosphatase is implicated in ovarian cancer growth. Clin Cancer Res 11: 6835–6839 doi:10.1158/1078-0432.CCR-04-2357 16203771 10.1158/1078-0432.CCR-04-2357

[33] Ren T, Jiang B, Xing X, Dong B, Peng L, et al (2009) Prognostic significance of phosphatase of regenerating liver-3 expression in ovarian cancer. Pathol Oncol Res 15: 555–560 doi:10.1007/s12253-009-9153-1 19247814 10.1007/s12253-009-9153-1

[34] Ma Y, Li B (2011) Expression of phosphatase of regenerating liver-3 in squamous cell carcinoma of the cervix. Med Oncol 28: 775–780 doi:10.1007/s12032-010-9514-3 20364335 10.1007/s12032-010-9514-3

[35] Wallin AR, Svanvik J, Adell G, Sun X-F (2006) Expression of PRL proteins at invasive margin of rectal cancers in relation to preoperative radiotherapy. Int J Radiat Oncol Biol Phys 65: 452–458 doi:10.1016/j.ijrobp.2005.12.043 16626893 10.1016/j.ijrobp.2005.12.043

[36] Zhou J, Wang S, Lu J, Li J, Ding Y (2009) Over-expression of phosphatase of regenerating liver-3 correlates with tumor progression and poor prognosis in nasopharyngeal carcinoma. Int J Cancer 124: 1879–1886 doi:10.1002/ijc.24096 19101992 10.1002/ijc.24096

[37] Liu Y-Q, Li H-X, Lou X, Lei J-Y (2008) Expression of phosphatase of regenerating liver 1 and 3 mRNA in esophageal squamous cell carcinoma. Arch Pathol Lab Med 132: 1307–1312 doi:10.1043/1543-2165(2008)132[1307:EOPORL]2.0.CO;2 18684031 10.5858/2008-132-1307-EOPORL

[38] Ooki A, Yamashita K, Kikuchi S, Sakuramoto S, Katada N, et al (2010) Phosphatase of regenerating liver-3 as a convergent therapeutic target for lymph node metastasis in esophageal squamous cell carcinoma. Int J Cancer 127: 543–554 doi:10.1002/ijc.25082 19960436 10.1002/ijc.25082

[39] Hassan NMM, Hamada J, Kameyama T, Tada M, Nakagawa K, et al (2011) Increased expression of the PRL-3 gene in human oral squamous cell carcinoma and dysplasia tissues. Asian Pac J Cancer Prev 12: 947–951. 21790231

[40] Reich R, Hadar S, Davidson B (2011) Expression and clinical role of protein of regenerating liver (PRL) phosphatases in ovarian carcinoma. Int J Mol Sci 12: 1133–1145 doi:10.3390/ijms12021133 21541048 10.3390/ijms12021133 PMC3083695

[41] Yamashita S, Masuda Y, Matsumoto K, Okumura Y, Matsuzaki H, et al (2007) Down-regulation of the human PRL-3 gene is associated with the metastasis of primary non-small cell lung cancer. Ann Thorac Cardiovasc Surg 13: 236–239. 17717498

[42] Hatate K, Yamashita K, Hirai K, Kumamoto H, Sato T, et al (2008) Liver metastasis of colorectal cancer by protein-tyrosine phosphatase type 4A, 3 (PRL-3) is mediated through lymph node metastasis and elevated serum tumor markers such as CEA and CA19-9. Oncol Rep 20: 737–743. 18813812

[43] Basak S, Jacobs SBR, Krieg AJ, Pathak N, Zeng Q, et al (2008) The metastasis-associated gene Prl-3 is a p53 target involved in cell-cycle regulation. Mol Cell 30: 303–314 doi:10.1016/j.molcel.2008.04.002 18471976 10.1016/j.molcel.2008.04.002 PMC3951836

[44] Diamond RH, Peters C, Jung SP, Greenbaum LE, Haber BA, et al (1996) Expression of PRL-1 nuclear PTPase is associated with proliferation in liver but with differentiation in intestine. Am J Physiol 271: G121–129. 8760115 10.1152/ajpgi.1996.271.1.G121

[45] Kong W, Swain GP, Li S, Diamond RH (2000) PRL-1 PTPase expression is developmentally regulated with tissue-specific patterns in epithelial tissues. Am J Physiol Gastrointest Liver Physiol 279: G613–621. 10960362 10.1152/ajpgi.2000.279.3.G613

[46] Forte E, Orsatti L, Talamo F, Barbato G, De Francesco R, et al (2008) Ezrin is a specific and direct target of protein tyrosine phosphatase PRL-3. Biochim Biophys Acta 1783: 334–344 doi:10.1016/j.bbamcr.2007.11.004 18078820 10.1016/j.bbamcr.2007.11.004

[47] Peters CS, Liang X, Li S, Kannan S, Peng Y, et al (2001) ATF-7, a novel bZIP protein, interacts with the PRL-1 protein-tyrosine phosphatase. J Biol Chem 276: 13718–13726 doi:10.1074/jbc.M011562200 11278933 10.1074/jbc.M011562200

[48] Fiordalisi JJ, Keller PJ, Cox AD (2006) PRL tyrosine phosphatases regulate rho family GTPases to promote invasion and motility. Cancer Res 66: 3153–3161 doi:10.1158/0008-5472.CAN-05-3116 16540666 10.1158/0008-5472.CAN-05-3116

[49] Zeng Q, Si X, Horstmann H, Xu Y, Hong W, et al (2000) Prenylation-dependent association of protein-tyrosine phosphatases PRL-1, -2, and -3 with the plasma membrane and the early endosome. J Biol Chem 275: 21444–21452 doi:10.1074/jbc.M000453200 10747914 10.1074/jbc.M000453200

[50] Sun J-P, Luo Y, Yu X, Wang W-Q, Zhou B, et al (2007) Phosphatase activity, trimerization, and the C-terminal polybasic region are all required for PRL1-mediated cell growth and migration. J Biol Chem 282: 29043–29051 doi:10.1074/jbc.M703537200 17656357 10.1074/jbc.M703537200

[51] Song R, Qian F, Li Y-P, Sheng X, Cao S-X, et al (2009) Phosphatase of regenerating liver-3 localizes to cyto-membrane and is required for B16F1 melanoma cell metastasis in vitro and in vivo. PLoS ONE 4: e4450 doi:10.1371/journal.pone.0004450 19214221 10.1371/journal.pone.0004450 PMC2635958

[52] Achiwa H, Lazo JS (2007) PRL-1 tyrosine phosphatase regulates c-Src levels, adherence, and invasion in human lung cancer cells. Cancer Res 67: 643–650 doi:10.1158/0008-5472.CAN-06-2436 17234774 10.1158/0008-5472.CAN-06-2436

[53] Luo Y, Liang F, Zhang Z-Y (2009) PRL1 promotes cell migration and invasion by increasing MMP2 and MMP9 expression through Src and ERK1/2 pathways. Biochemistry 48: 1838–1846 doi:10.1021/bi8020789 19199380 10.1021/bi8020789 PMC2765538

[54] Liang F, Luo Y, Dong Y, Walls CD, Liang J, et al (2008) Translational control of C-terminal Src kinase (Csk) expression by PRL3 phosphatase. J Biol Chem 283: 10339–10346 doi:10.1074/jbc.M708285200 18268019 10.1074/jbc.M708285200 PMC2447647

[55] Wang H, Quah SY, Dong JM, Manser E, Tang JP, et al (2007) PRL-3 down-regulates PTEN expression and signals through PI3K to promote epithelial-mesenchymal transition. Cancer Res 67: 2922–2926 doi:10.1158/0008-5472.CAN-06-3598 17409395 10.1158/0008-5472.CAN-06-3598

[56] Parcellier A, Tintignac LA, Zhuravleva E, Hemmings BA (2008) PKB and the mitochondria: AKTing on apoptosis. Cell Signal 20: 21–30 doi:10.1016/j.cellsig.2007.07.010 17716864 10.1016/j.cellsig.2007.07.010

[57] Stambolic V, Woodgett JR (2006) Functional distinctions of protein kinase B/Akt isoforms defined by their influence on cell migration. Trends Cell Biol 16: 461–466 doi:10.1016/j.tcb.2006.07.001 16870447 10.1016/j.tcb.2006.07.001

[58] Xu T, Rubin GM (1993) Analysis of genetic mosaics in developing and adult Drosophila tissues. Development 117: 1223–1237. 8404527 10.1242/dev.117.4.1223

[59] Neufeld TP, de la Cruz AF, Johnston LA, Edgar BA (1998) Coordination of growth and cell division in the Drosophila wing. Cell 93: 1183–1193. 9657151 10.1016/s0092-8674(00)81462-2

[60] Jeong DG, Kim SJ, Kim JH, Son JH, Park MR, et al (2005) Trimeric structure of PRL-1 phosphatase reveals an active enzyme conformation and regulation mechanisms. J Mol Biol 345: 401–413 doi:10.1016/j.jmb.2004.10.061 15571731 10.1016/j.jmb.2004.10.061

[61] Vidal M, Warner S, Read R, Cagan RL (2007) Differing Src signaling levels have distinct outcomes in Drosophila. Cancer Res 67: 10278–10285 doi:10.1158/0008-5472.CAN-07-1376 17974969 10.1158/0008-5472.CAN-07-1376 PMC2892182

[62] Kozlov G, Cheng J, Ziomek E, Banville D, Gehring K, et al (2004) Structural Insights into Molecular Function of the Metastasis-associated Phosphatase PRL-3. J Biol Chem 279: 11882–11889 doi:10.1074/jbc.M312905200 14704153 10.1074/jbc.M312905200

[63] Sun J-P, Wang W-Q, Yang H, Liu S, Liang F, et al (2005) Structure and biochemical properties of PRL-1, a phosphatase implicated in cell growth, differentiation, and tumor invasion. Biochemistry 44: 12009–12021 doi:10.1021/bi0509191 16142898 10.1021/bi0509191

[64] Yu L, Kelly U, Ebright JN, Malek G, Saloupis P, et al (2007) Oxidative stress-induced expression and modulation of Phosphatase of Regenerating Liver-1 (PRL-1) in mammalian retina. Biochim Biophys Acta 1773: 1473–1482 doi:10.1016/j.bbamcr.2007.06.005 17673310 10.1016/j.bbamcr.2007.06.005 PMC2118714

[65] Skinner AL, Vartia AA, Williams TD, Laurence JS (2009) Enzyme activity of phosphatase of regenerating liver is controlled by the redox environment and its C-terminal residues. Biochemistry 48: 4262–4272 doi:10.1021/bi900241k 19341304 10.1021/bi900241k PMC2759777

[66] Min S-H, Kim DM, Heo Y-S, Kim Y-I, Kim HM, et al (2009) New p53 target, phosphatase of regenerating liver 1 (PRL-1) downregulates p53. Oncogene 28: 545–554 doi:10.1038/onc.2008.409 18997816 10.1038/onc.2008.409

[67] Min S-H, Kim DM, Heo Y-S, Kim HM, Kim I-C, et al (2010) Downregulation of p53 by phosphatase of regenerating liver 3 is mediated by MDM2 and PIRH2. Life Sci 86: 66–72 doi:10.1016/j.lfs.2009.11.010 19945467 10.1016/j.lfs.2009.11.010

[68] Rudrapatna VA, Cagan RL, Das TK (2012) Drosophila cancer models. Dev Dyn 241: 107–118 doi:10.1002/dvdy.22771 22038952 10.1002/dvdy.22771 PMC3677821

[69] Sanson B, White P, Vincent JP (1996) Uncoupling cadherin-based adhesion from wingless signalling in Drosophila. Nature 383: 627–630 doi:10.1038/383627a0 8857539 10.1038/383627a0

[70] O'Reilly AM, Ballew AC, Miyazawa B, Stocker H, Hafen E, et al (2006) Csk differentially regulates Src64 during distinct morphological events in Drosophila germ cells. Development 133: 2627–2638 doi:10.1242/dev.02423 16775001 10.1242/dev.02423

