# Supplementary figures and images for: Drosophila PRL-1 Is a Growth Inhibitor That Counteracts the Function of the Src Oncogene
Source: PLoS One. 2013 Apr 8;8(4):e61084. doi: 10.1371/journal.pone.0061084 (PMC3620046; doi:10.1371/journal.pone.0061084)

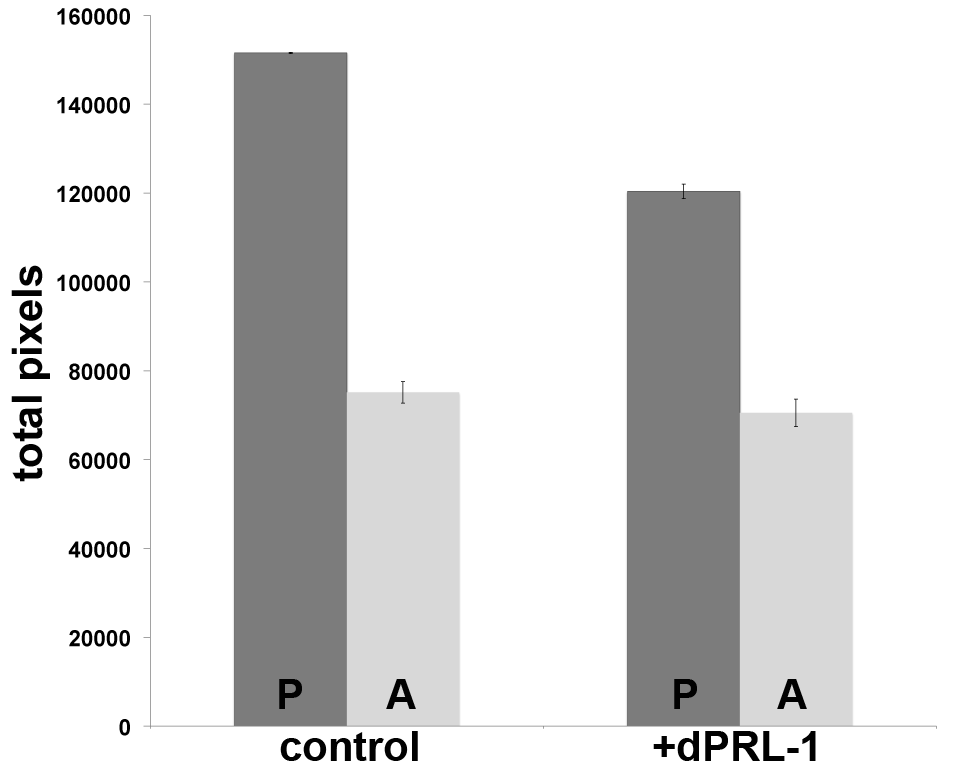

Supplement: Figure S1 — Quantification of growth inhibition following dPRL-1 expression in the wing. Comparison of surface area in the posterior (P) and anterior (A) compartments of adult wings of animals expressing dPRL-1 (w; enGal4, UAS-dPRL-1) compared to control (w; enGal4; +). dPRL-1 reduces the area of the posterior compartment by 20% (p = 003). The small reduction in the anterior compartment was not statistically significant (p = 0.24). Data is presented as average +/− standard error. (TIF) [file pone.0061084.s001.tif]
